# Levothyroxine Therapy and Predictors of Cardiovascular Risk in Clinical Hypothyroidism: A Prospective Cohort Study

**DOI:** 10.7759/cureus.30969

**Published:** 2022-11-01

**Authors:** Rituparna Maiti, Rashmi R Mohanty, Archana Mishra, Anupam Dey, Nishant Verma

**Affiliations:** 1 Pharmacology, All India Institute of Medical Sciences, Bhubaneswar, IND; 2 General Medicine, All India Institute of Medical Sciences, Bhubaneswar, IND

**Keywords:** hscrp, zulewski score, cardiovascular risk, insulin resistance, adiponectin, hypothyroidism

## Abstract

Background

Hypothyroidism is associated with hypoadiponectinemia, insulin resistance, and increased cardiovascular risk. The association of adiponectin, insulin resistance, and future cardiovascular risk in clinical hypothyroidism and the effect of levothyroxine are non-conclusive because of the contradictory results. The present prospective cohort study has been conducted to evaluate the effect of levothyroxine on serum adiponectin, insulin resistance, and cardiovascular risk in patients with clinical hypothyroidism.

Methods

Sixty patients with clinical hypothyroidism who were prescribed levothyroxine were recruited following selection criteria and changes in Zulewski’s score, glycemic parameters, homeostatic model assessment of insulin resistance (HOMA-IR), quantitative insulin sensitivity check index (QUICKI), lipid profile, serum adiponectin, serum high-sensitivity C-reactive protein (hs-CRP), cardiovascular risk indices, and Framingham risk score were assessed 12 weeks post-levothyroxine therapy. Receiver operating characteristic (ROC) analysis was done to detect the cut-off for adiponectin levels to differentiate between responders and non-responders. Neural network models were created to predict the risk of cardiovascular morbidity.

Results

Post-levothyroxine therapy, there was a significant improvement in body mass index (BMI) (p = 0.025), diastolic blood pressure (p = 0.021), Zulewski’s score (p < 0.001), serum insulin (p = 0.005), fasting sugar (p < 0.001), serum adiponectin (p < 0.001), thyroid profile (p < 0.001), total cholesterol (p < 0.001), low-density lipoprotein (p < 0.001), high-density lipoprotein (p = 0.007), triglycerides (p = 0.002), and subcutaneous fat (p = 0.015). Serum adiponectin showed significant improvement in hypothyroid patients compared to euthyroid individuals (mean difference: -2.21; 95% CI: -2.52 to -1.91; p < 0.001). Mean difference in insulin resistance (HOMA-IR: 0.3, p < 0.001; QUICKI: -0.002, p < 0.001) and cardiovascular risk (atherogenic index: 0.12, p = 0.04; coronary risk index: 0.14, p = 0.038; Framingham risk score: 0.65, p = 0.041) also showed improvement. Serum adiponectin and thyroid-stimulating hormone levels were directly correlated with insulin resistance and cardiovascular risk scores.

Conclusion

The reduced serum adiponectin level and increased cardiovascular risk in clinical hypothyroidism were improved with hormone replacement, and serum adiponectin level was found to be a good prognostic marker for the treatment response.

## Introduction

Clinical or overt hypothyroidism is associated with an increased risk of atherosclerosis and coronary artery diseases [1]. Long-term clinical hypothyroidism leads to serious cardiovascular manifestations, including decreased intravascular volume, increased systemic vascular resistance, and hypertension [2]. Hypothyroidism is one of the leading causes of secondary dyslipidemia. The common dyslipidemic changes of hypothyroidism are raised low-density lipoprotein (LDL), very low-density lipoprotein (VLDL), and apolipoprotein A [3,4]. Other than dyslipidemia, hemodynamic changes, endothelial dysfunction, and metabolic changes are important contributing factors to cardiovascular risk [5]. Metabolic syndrome and insulin resistance also contribute to existing risk, as insulin-resistant patients with elevated thyroid-stimulating hormone (TSH) have higher LDL levels [6].

From the previously published literature, it is evident that serum adiponectin is inversely related to insulin resistance, and a higher level of serum adiponectin is considered cardioprotective [7-10]. Hypoadiponectinemia is associated with insulin resistance, obesity, type 2 diabetes mellitus, hypertension, and coronary artery disease [11-13].

Levothyroxine has proven hypolipidemic and antioxidant properties and has a beneficial effect on cardiovascular function and lipid profile. However, the association of insulin resistance and serum adiponectin in clinical or overt hypothyroidism and future cardiovascular risk is still not conclusive because of the paucity of clinical studies and contradictory results from some of the studies [14-18]. This study was conducted with the objective to evaluate serum adiponectin, insulin resistance, cardiovascular risk, and their correlation (if any) in patients with clinical or overt hypothyroidism and to evaluate the effect of levothyroxine on these parameters.

## Materials and methods

Study design

This prospective cohort study was conducted in a single center following the ethical guidelines for biomedical research on human subjects from the Indian Council of Medical Research (ICMR). The present study was conducted after obtaining written approval from the Institutional Ethics Committee of All India Institute of Medical Sciences, Bhubaneswar (T/EM-F/Pharm/14/03).

Study population

Patients of either sex aged 18 years or above suffering from clinical hypothyroidism and requiring treatment (TSH levels > 10 µIU/mL) were included in the study. All recruited patients were free from renal/hepatic disorders, diabetes mellitus, and chronic inflammatory diseases and were not taking any medications for thyroid dysfunction. The euthyroid subjects did not have any significant medical or surgical disease. The patients with other comorbidities that can interfere with the outcome measures, patients who were on levothyroxine therapy or taking other medications, patients with subacute thyroiditis, and pregnant and lactating mothers were excluded from the study.

Study procedure and data collection

The present study was a 12-week prospective cohort study conducted on patients suffering from clinical hypothyroidism. Sixty patients suffering from clinical hypothyroidism attending the outpatient department of general medicine of our institute were recruited for the present study following selection criteria. Sixty age and sex-matched euthyroid subjects served as the comparator group. Patients with clinical hypothyroidism were evaluated at baseline and again after 12 weeks following levothyroxine therapy. The euthyroid subjects were evaluated once at baseline and after 12 weeks. At the first visit, after taking a detailed history including baseline symptomatology, clinical evaluation (anthropometry, blood pressure, Zulewski's clinical score for hypothyroidism, metabolic syndrome score, and Framingham cardiovascular risk score), laboratory investigation (thyroid profile, lipid profile, blood sugar, serum adiponectin, serum high-sensitivity C-reactive protein (hs-CRP), serum insulin, homeostatic model assessment of insulin resistance (HOMA-IR), and quantitative insulin sensitivity check index (QUICKI)), and bioelectrical impedance analysis for body fat mass were done, and treatment was started with levothyroxine. The dosage of levothyroxine (LT4) was adjusted (at the fourth and eighth-week follow-up) to achieve free T4 (FT4) and TSH levels within the normal range. After 12 weeks, all the subjects were followed up for repetition of clinical and laboratory tests.

Outcome measures

The parameters evaluated in the present study are anthropometric parameters such as height, weight, body mass index (BMI), and abdominal circumference; blood pressure (both systolic and diastolic); Zulewski's clinical scoring for hypothyroidism; metabolic syndrome diagnostic scoring; biochemical tests such as thyroid function test (TSH, free T3, FT4), lipid profile, fasting blood sugar, serum insulin, and serum adiponectin; assessment of HOMA-IR and QUICKI; assessment of cardiovascular risk by cardiovascular risk assessment scoring (Framingham scoring); and cardiovascular risk indices such as coronary risk index (CRI), atherogenic index (AI), cardiovascular risk index (CVRI), serum hs-CRP.

Statistical analysis

Continuous data have been presented as a mean ± standard deviation (SD) and categorical data as percentages. Comparison of means between the study groups was performed using t-test/Mann-Whitney U test and within the group by two-sided paired t-test/Wilcoxon signed rank test. Fisher’s exact test was used for comparing categorical variables between the groups. Pearson product-moment correlation coefficient was calculated to measure the correlation between outcome measures. Regression analysis was done to predict the effect of various variables on serum adiponectin, creating the best fit model. Receiver operating characteristic (ROC) analysis was performed to detect the cut-off value for the change in adiponectin levels to differentiate the responders and non-responders based on a 70% reduction in TSH levels. A multilayer perceptron model was created to predict the risk of cardiovascular morbidity depending on various covariates. Statistical analyses were performed using statistical software SPSS 20.0 (IBM Corp., Armonk, NY), considering a significance level of p < 0.05.

## Results

Patient demographics and baseline characteristics

**Figure 1:**
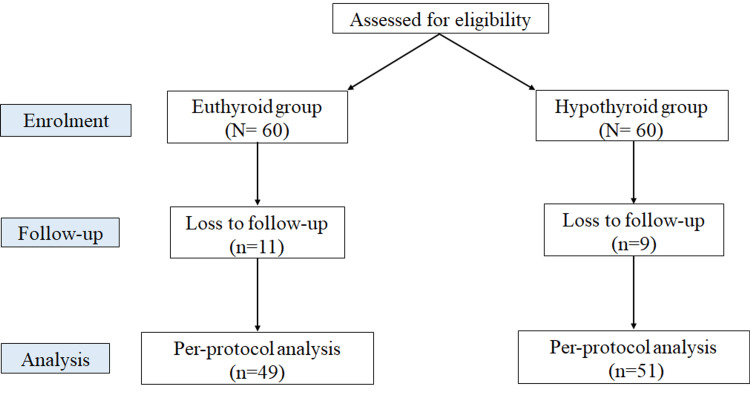
The flow of participants through each stage of the study "N" and "n" denote the number of patients.

Out of 60 hypothyroid patients recruited, 51 patients completed the follow-up, and out of 60 euthyroid subjects, 49 completed the follow-up (Figure 1). Out of nine follow-up loss among hypothyroid patients, four patients were excluded because the anti-hypertensive medication was started, and the reason for loss for the other five patients is unknown. The mean age of the hypothyroid group was 41.5 years and of the euthyroid group was 40.2 years. In both groups, 19 patients were male, and 41 were female. The baseline demographic and clinical data of the hypothyroid group have been presented in Table 1.

**Table 1:** Baseline demographic and clinical parameters All values are represented as mean ± standard deviation. BMI, body mass index; hs-CRP, high-sensitivity C-reactive protein; TSH, thyroid-stimulating hormone; FT3, free T3; FT4, free T4; LDL-C, low-density lipoprotein cholesterol; HDL-C, high-density lipoprotein cholesterol; VLDL-C, very low-density lipoprotein cholesterol.

Parameters	Hypothyroid group (N = 60)
Gender (male:female)	19:41
Age (years)	41.5 ± 11.5
Height (cm)	157.7 ± 8.22
Weight (kg)	63.63 ± 13.17
BMI (kg/m^2^)	25.5 ± 4.5
Waist circumference (inches)	36.72 ± 3.83
Systolic blood pressure (mm of Hg)	128.72 ± 17.86
Diastolic blood pressure (mm of Hg)	78.28 ± 10.32
Serum hs-CRP (mg/L)	1.49 ± 0.47
Insulin (mIU/L)	15.29 ± 4.60
Fasting blood sugar (mmol/L)	4.64 ± 0.65
Serum adiponectin levels (ng/mL)	8.04 ± 2.16
Serum TSH (mIU/L)	14.43 ± 11.17
Serum FT3 (pmol/L)	2.45 ± 0.56
Serum FT4 (pmol/L)	7.67 ± 1.77
Serum total cholesterol (mg/dL)	236.07 ± 37.18
Serum LDL-C (mg/dL)	157.45 ± 30.35
Serum HDL-C (mg/dL)	45.05 ± 8.54
Serum VLDL-C (mg/dL)	33.57 ± 16.26
Serum triglycerides (mg/dL)	155.83 ± 46.90
Visceral fat (%)	8.19 ± 4.51
Subcutaneous fat (%)	24.53 ± 7.46

Change in anthropometric parameters

After 12 weeks of therapy with levothyroxine, there was a significant decrease in body weight (1.06; 95% CI: 0.19 to 1.92; p = 0.017), BMI (0.37; 95% CI: 0.048 to 0.698; p = 0.025), and waist circumference (0.43; 95% CI: 0.15 to 0.69; p = 0.003) in the hypothyroid group (Table 2). There was no significant change in anthropometric parameters among euthyroid subjects.

**Table 2:** Comparison of pre and post-levothyroxine therapy All parameters are expressed in mean ± SD (except Framingham risk score). ^a^ Median and interquartile range. ^b^ Paired t-test/Wilcoxon signed rank test. BMI, body mass index; hs-CRP, high-sensitivity C-reactive protein; TSH, thyroid-stimulating hormone; LDL, low-density lipoprotein; HDL, high-density lipoprotein; VLDL, very low-density lipoprotein cholesterol; HOMA-IR, homeostatic model assessment of insulin resistance; QUICKI, quantitative insulin sensitivity check index.

Parameters	1^st^ visit (n = 51)	2^nd^ visit (n = 51)	Mean difference (95% CI)	P-value ^b^
Weight (kg)	63.40 ± 13.39	62.34 ± 12.52	1.06 (0.19 to 1.92)	0.017
BMI (kg/m^2^)	25.29 ± 4.39	24.92 ± 4.36	0.37 (0.048 to 0.698)	0.025
Waist circumference (inches)	36.57 ± 3.90	36.14 ± 3.57	0.43 (0.15 to 0.69)	0.003
Systolic blood pressure (mmHg)	128.78 ± 18.44	127.82 ± 17.71	0.96 (-1.94 to 3.86)	0.509
Diastolic blood pressure (mmHg)	77.63 ± 10.37	75.04 ± 11.09	2.59 (0.41 to 4.76)	0.021
Total Zulewski score	7.62 ± 1.93	2.88 ± 2.31	4.74 (3.96 to 5.50)	<0.001
Serum insulin (mIU/L)	15.26 ± 4.42	14.23 ± 4.87	1.03 (0.32 to 1.74)	0.005
Fasting blood sugar (mMol/L)	4.67 ± 0.68	4.47 ± 0.65	0.20 (0.16 to 0.24)	<0.001
Adiponectin (ng/mL)	8.05 ± 2.04	10.13 ± 2.35	-2.08 (-2.34 to -1.82)	<0.001
Free T3 (pmol/L)	2.45 ± 0.54	2.77 ± 0.76	-0.32 (-0.51 to -0.12)	0.002
Free T4 (pmol/L)	7.68 ± 1.69	9.60 ± 2.12	-1.92 (-2.04 to -1.80)	<0.001
TSH (mIU/L)	14.43 ± 11.27	8.03 ± 5.67	6.39 (4.86 to 7.93)	<0.001
Total cholesterol (mg/dL)	236.57 ± 39.07	224.68 ± 38.57	11.89 (7.97 to 15.81)	<0.001
LDL (mg/dL)	158.04 ± 31.39	148.48 ± 32.74	9.56 (5.82 to 13.30)	<0.001
HDL (mg/dL)	44.67 ± 8.17	43.45 ± 7.75	1.22 (0.34 to 2.09)	0.007
VLDL (mg/dL)	33.86 ± 17.11	32.75 ± 16.13	1.11 (0.26 to 1.96)	0.011
Triglycerides (mg/dL)	156.53 ± 42.33	150.41 ± 37.71	6.12 (2.30 to 9.30)	0.002
Visceral fat (%)	8.57 ± 4.68	8.27 ± 4.52	0.29 (-0.17 to 0.76)	0.215
Subcutaneous fat (%)	24.75 ± 7.70	24.00 ± 7.36	0.75 (0.15 to 1.35)	0.015
HOMA-IR	3.21 ± 1.13	2.87 ± 1.16	0.34 (0.18 to 0.49)	<0.001
QUICKI	0.55 ± 0.06	0.57 ± 0.06	-0.02 (-0.03 to -0.01)	<0.001
Atherogenic index	3.63 ± 0.92	3.51 ± 0.95	0.12 (0.01 to 0.24)	0.041
Coronary risk index	5.40 ± 1.06	5.26 ± 1.03	0.14 (0.01 to 0.26)	0.038
Cardiovascular risk index	3.60 ± 1.19	3.57 ± 1.11	0.03 (-0.07 to 0.16)	0.515
Serum hs-CRP (mg/L)	1.50 ± 0.44	1.45 ± 0.42	0.05 (0.01 to 0.09)	0.013
Framingham risk score (%) ^a^	2 (1-8)	2 (1-8)	-	0.041
Framingham risk score (%)	6.20 ± 8.05	5.55 ± 7.84	0.65 (0.016 to 1.28)	0.045

Change in blood pressure

In the hypothyroid group, there was no significant change in systolic blood pressure (0.96, 95% CI: -1.94 to 3.86; p = 0.509), but diastolic blood pressure was significantly reduced (2.59; 95% CI: 0.41 to 4.76; p = 0.021) (Table 2). There was no significant change in blood pressure among euthyroid subjects.

Change in Zulewski score

After 12 weeks of therapy with levothyroxine, in the hypothyroid group, there was a clinical improvement in hypothyroidism in terms of a decrease in Zulewski score from 7.62 ± 1.93 to 2.88 ± 2.31 (95% CI: 3.96 to 5.50, p < 0.001) (Table 2).

Change in metabolic syndrome scoring

According to metabolic syndrome scoring, among hypothyroid patients, 36 patients (out of 51) were diagnosed as having metabolic syndrome, whereas, after 12 weeks of therapy with levothyroxine, 25 patients (out of 51) were diagnosed with metabolic syndrome. These data were put in the 2 x 2 contingency table and analyzed by Fischer’s exact test, and the change was found to be statistically significant (RR = 1.44; 95% CI: 1.034 to 2.006; p = 0.04).

Change in lipid profile

After 12 weeks of therapy with levothyroxine, in the hypothyroid group, there was a significant decrease in total cholesterol (11.89; 95% CI: 7.97 to 15.81; p < 0.001), LDL cholesterol (9.56; 95% CI: 5.82 to 13.30; p < 0.001), high-density lipoprotein (HDL) cholesterol (1.22; 95% CI: 0.34 to 2.09; p = 0.007), VLDL cholesterol (1.11; 95% CI: 0.26 to 1.96; p = 0.011), and serum triglyceride (6.12; 95% CI: 2.30 to 9.30; p = 0.002) (Table 2). There was no significant change in lipid profile among euthyroid subjects.

Change in thyroid function test

After 12 weeks of therapy with levothyroxine, in the hypothyroid group, there was a significant decrease in TSH (6.39; 95% CI: 4.86 to 7.93; p < 0.001), a significant increase in free T3 (-0.32; 95% CI: -0.51 to -0.12; p < 0.001), and a significant increase in free T4 (-1.92; 95% CI: -2.04 to -1.80; p < 0.001) (Table 2). There was no significant change in thyroid profile among euthyroid subjects.

Change in glycemic parameters

After 12 weeks of therapy with levothyroxine, in the hypothyroid group, there was a significant decrease in fasting blood sugar (0.20; 95% CI: 0.16 to 0.24; p < 0.001) and a significant reduction in fasting insulin level (1.03; 95% CI: 0.32 to 1.74; p = 0.005) (Table 2).

Change in insulin resistance

Insulin resistance was assessed by HOMA-IR and QUICKI. After 12 weeks of therapy with levothyroxine, in the hypothyroid group, there was a significant decrease in HOMA-IR (0.34; 95% CI: 0.18 to 0.49; p < 0.001) and an increase in QUICKI (-0.02; 95% CI: -0.03 to 0.01; p < 0.001) (Table 2).

Change in body fat distribution

After 12 weeks of therapy with levothyroxine, in the hypothyroid group, there was a significant decrease in subcutaneous fat (0.75; 95% CI: 0.15 to 1.35; p = 0.015); however, there was no significant change in visceral fat (0.29; 95% CI: -0.17 to 0.76; p = 0.215) (Table 2). There was no significant change in fat distribution among euthyroid subjects.

Change in hs-CRP

Serum hs-CRP decreased significantly from 1.50 ± 0.44 to 1.45 ± 0.42 (0.05; 95% CI: 0.01 to 0.09; p = 0.013) in the hypothyroid group over a period of 12 weeks (Table 2). There was no significant change in serum hs-CRP among euthyroid subjects.

Change in Framingham risk scoring

Because of wide variability and non-normal distribution, the Framingham score has been presented in the median and interquartile range. Though at baseline and after 12 weeks, median values were the same, Wilcoxon signed rank test indicated that the baseline score was significantly higher than the follow-up score (z = 96.5, p = 0.041) (Table 2).

Change in cardiovascular indices

The cardiovascular indices, which are derived parameters from the lipid profile, were found to be improved with levothyroxine therapy. There was a significant decrease in atherogenic index (0.12; 95% CI: 0.01 to 0.24; p = 0.041) and coronary risk index (0.14; 95% CI: 0.01 to 0.26; p = 0.038) over 12 weeks of therapy. There was no significant change in the cardiovascular risk index (0.03; 95% CI: -0.07 to 0.16; p = 0.515) (Table 2).

Change in serum adiponectin

**Figure 2:**
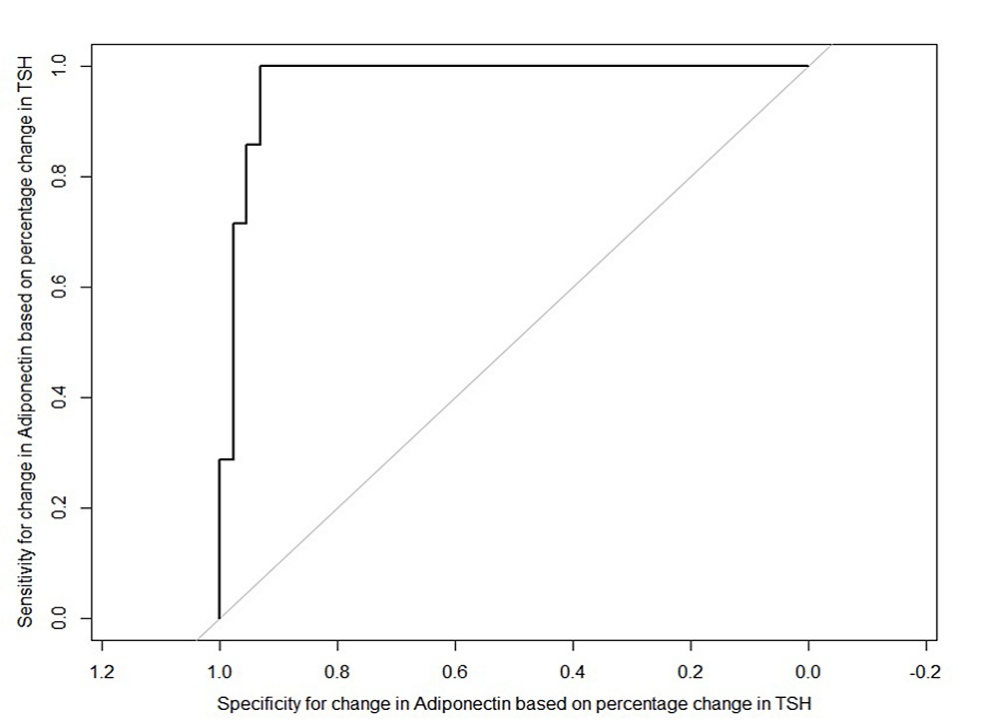
ROC curve for change in adiponectin to differentiate responders from non-responders based on the percentage change in TSH ROC, receiver operating characteristic; TSH, thyroid-stimulating hormone.

In the euthyroid group, there was no significant change in serum adiponectin level (0.016; 95% CI: 0.002 to 0.314; p = 0.098). In the hypothyroid group, serum adiponectin level was significantly lowered at baseline (8.04 ± 2.16 vs. 12.38 ± 2.95; 95% CI: 3.39 to 5.27; p < 0.001) in comparison to the euthyroid group. After 12 weeks of therapy with levothyroxine, in the hypothyroid group, there was a significant increase in serum adiponectin (-2.08; 95% CI: -2.34 to -1.82; p < 0.001). There was no significant change in serum adiponectin among euthyroid subjects. The mean changes of both the groups were compared and found to be statistically significant (-2.21, 95% CI: -2.52 to -1.91; p < 0.001) (Table 3). The cut-off value for the change in serum adiponectin with ROC analysis (area under the curve = 0.974) to differentiate responders from non-responders based on the percentage change in TSH was 5.895 ng/ml. The sensitivity and specificity of the cut-off value are 100% and 93.2%, respectively (Figure 2).

**Table 3:** Comparative change in serum adiponectin between euthyroid and hypothyroid group All values are represented as mean ± standard deviation. ^a^ Paired t-test. ​​​​​​​^b^ Unpaired t-test.

Variable	Hypothyroid group (n = 51)				Euthyroid group (n = 49)				Difference between groups ΔControl vs. ΔTest	
	Baseline	Follow-up	Mean diff Δ (95% CI)	P-value ^a^	Baseline	Follow-up	Mean diff Δ (95% CI)	P-value ^a^	Mean diff Δ (95% CI)	P-value ^b^
Serum adiponectin (ng/mL)	8.05 ± 2.04	10.13 ± 2.35	-2.08 (-2.34 to -1.82)	<0.001	12.54 ± 2.81	12.41 ± 2.68	0.016 (0.002 to 0.314)	0.098	-2.21 (-2.52 to -1.91)	<0.001

Correlation analysis

The correlations between TSH, adiponectin, and cardiovascular indices were found to be positively correlated and statistically significant (Table 4). TSH and adiponectin levels are inversely correlated to each other (r = -0.759, p < 0.001). TSH and adiponectin are both correlated to indices of insulin resistance, namely, HOMA-IR and QUICKI (Table 5).

**Table 4:** Correlation between thyroid-stimulating hormone (TSH), adiponectin, and cardiovascular risk indices

	Atherogenic index		Coronary risk index		Cardiovascular risk index		Framingham risk score	
	Correlation coefficient (r)	P-value	Correlation coefficient (r)	P-value	Correlation coefficient (r)	P-value	Correlation coefficient (r)	P-value
Serum TSH (mIU/L)	0.308	0.009	0.325	0.011	0.294	0.023	0.173	0.185
Serum adiponectin (ng/mL)	0.421	0.001	0.409	0.001	0.302	0.019	0.269	0.038

**Table 5:** Correlation between TSH, adiponectin, and Insulin resistance TSH, thyroid-stimulating hormone; HOMA-IR, homeostatic model assessment of insulin resistance; QUICKI, quantitative insulin sensitivity check index.

	HOMA-IR		QUICKI	
	Correlation coefficient (r)	P-value	Correlation coefficient (r)	P-value
Serum adiponectin (ng/mL)	-0.807	<0.001	0.795	<0.001
Serum TSH (mIU/L)	0.629	<0.001	-0.490	<0.001

Regression analysis

A linear regression analysis was done, and the best fit model was selected to predict serum adiponectin levels at baseline based on age, sex, BMI, fasting blood glucose, TSH, T3, visceral fat, subcutaneous fat, and waist circumference. The equation calculated was found to be significant with F (9,51) = 33.43 (p < 0.001), with R^2^ = 0.86. It was found that when adjusted for all independent variables, the change in serum TSH, insulin, fasting blood glucose, subcutaneous fat, and age can predict serum adiponectin levels. The standardized beta coefficient and p-value for the independent variables are mentioned in Table 6.

**Table 6:** Standardized beta coefficients and p-value for independent variables with serum adiponectin as the dependent variable TSH, thyroid-stimulating hormone; FT3, free T3.

Independent variables	Standardized β coefficients	T	P-value
Sex	0.164	2.111	0.040
Age	-0.125	-2.189	0.033
Insulin	-0.285	-3.669	0.001
Fasting blood glucose	-0.250	-4.031	<0.001
TSH	-0.400	-5.624	<0.001
FT3	0.002	0.033	0.974
Visceral fat	-0.001	-0.018	0.985
Subcutaneous fat	-0.347	-3.818	<0.001
Waist circumference	0.114	1.685	0.09

Artificial neural network model for predicting cardiac morbidity

**Figure 3:**
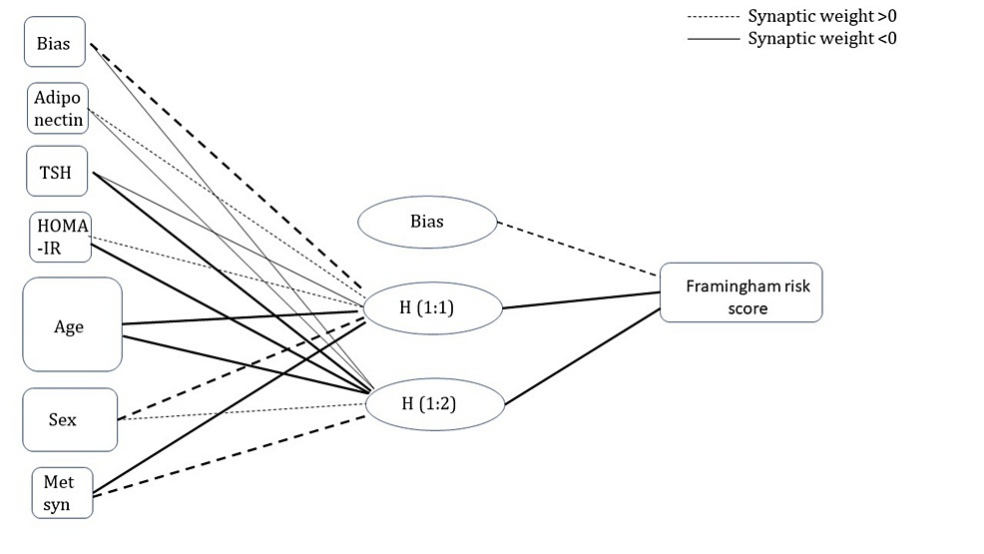
Neural network for predicting Framingham risk score based on covariates with input layer (age, gender, serum TSH, HOMA-IR, metabolic syndrome, serum adiponectin), one hidden layer with two neurons, and output layer as Framingham risk score Dashed lines represent synaptic weight > 0 and solid lines represent synaptic weight < 0; intensity varies with weight. The size of the boxes in the output layer represents the importance of the covariate in predicting the output. TSH, thyroid-stimulating hormone; HOMA-IR, homeostatic model assessment of insulin resistance.

Multilayer perceptron modeling was done to form a model to predict cardiac morbidity as represented by the Framingham risk score. The covariates that are known to be predictors of cardiovascular health were chosen as covariates, namely, age, gender, serum TSH, serum adiponectin, insulin resistance (as calculated by HOMA-IR), and metabolic syndrome score. The cases assigned to training and testing were 49:11 following a 90:10 partition (due to the smaller sample size). The model obtained contains one layer of hidden neurons with two units in the layer. The relative error in training was 0.60%, whereas, in testing, it was 0.13%. Parameter estimates for prediction have been depicted in Table 7. Of all the covariates used, age was found to be the most important contributor, and metabolic syndrome and serum adiponectin as the least important contributors (age: 100%; gender: 75.5%; serum TSH: 24.1%; HOMA-IR: 17%; serum adiponectin: 11%; metabolic syndrome score: 10.8%) to the model. Bias terms have been incorporated in input and hidden layers to make the model best fit (Figure 3).

**Table 7:** Parameter estimates for the neural network model TSH, thyroid-stimulating hormone; HOMA-IR, homeostatic model assessment of insulin resistance.

Covariates (predictors)		Predicted		
		Hidden layer		Output layer
		H (1:1)	H (1:2)	Framingham risk score
Input layer	Age	-1.103	-0.538	
	Sex	0.748	0.414	
	Serum TSH	-0.131	-0.190	
	HOMA-IR	0.233	-0.244	
	Serum adiponectin	0.408	-0.156	
	Metabolic syndrome	-0.417	0.143	
	Bias	1.166	-0.073	
Output layer				
	H (1:1)			-1.266
	H (1:2)			-1.184
	Bias			0.498

## Discussion

In the present study, we have evaluated the effect of levothyroxine therapy on thyroid hormonal profile, insulin resistance, and cardiovascular risk indices. A three-month therapy with levothyroxine had a significant effect on anthropometric measurements, which was evident from the reduction in weight, BMI, and waist circumference. There was a reduction in both systolic and diastolic blood pressure. We observed a trend toward normalization of the thyroid profile with an increase in T3 and T4 and a decrease in TSH levels. Levothyroxine had an overall beneficial effect on lipid profile showing a reduction in LDL, VLDL, triglycerides, and total cholesterol, though the treatment caused a decrease in HDL as well. There was a decrease in the percentage of patients with metabolic syndrome and a reduction in insulin resistance measured by HOMA-IR and QUICKI over 12 weeks of therapy. A decrease in fasting blood glucose and serum insulin levels was also recorded in this study. There was a decrease in subcutaneous fat after treatment; however, the reduction in visceral fat percentage was not significant with treatment. In this study, we have observed a direct correlation between serum TSH levels and insulin resistance and the risk of cardiovascular morbidity. There was an inverse association between serum TSH levels and adiponectin, i.e., serum adiponectin levels decreased with increasing severity of the disease. In our study, serum adiponectin levels increased with levothyroxine replacement therapy. Insulin sensitivity was also found to be improved with treatment over 12 weeks.

Thyroid hormone influences both synthesis and degradation of lipids, with evidence of the latter being more significantly affected than the former, principally by enhancing the utilization of lipid substrates, increasing lipoprotein lipase activity, and increasing expression of LDL receptors [19,20]. Several indices have been optimized to enhance the predictive capacity of lipid profile panels for future cardiovascular events. The ratios have better predictive value than independent values of lipoproteins [21]. The risk indices had a direct correlation with serum TSH and adiponectin levels at baseline. However, levothyroxine replacement therapy for 12 weeks had a significant corrective effect on insulin sensitivity and cardiac risk indices such as atherogenic index, coronary risk index, and Framingham risk score. It has been put forward earlier that hypothyroidism increases the risk of atherogenesis and coronary artery diseases through various mechanisms like a decreased expression of Ca^2+^-ATPase in sarcoplasmic reticulum or disinhibition of ATPase by increased expression of phospholamban or modulation of the renin-angiotensin-aldosterone axis [22]. Thyroid replacement therapy has been shown to have a beneficial effect on cardiac function parameters supporting findings from our study that restoration of thyroid profile toward euthyroid states most often reverses abnormal cardiovascular hemodynamics [23].

Consistent with other studies, our study also found an inherent relation between thyroid function and insulin resistance [24]. Though our study calculated insulin resistance by HOMA-IR and QUICKI, an improvement was observed in insulin sensitivity, as found by Deyneli et al. (2014), who studied the effect of levothyroxine therapy on insulin resistance by euglycemic hyperinsulinemic clamp technique and found there was an improvement in sensitivity with levothyroxine therapy [25]. It might be due to the positive transcriptional regulation of glucose transporter type 4 (GLUT4) in muscle by thyroid hormone, an important component of glucose metabolism. In a previous study by Altinova et al. (2006), there was no significant association between adiponectin and TSH levels [17]. It has been proposed by Fernández-Real et al. (2003) that adiponectin, along with thyroid hormones, also has an important role in insulin resistance [26]. Our study has found insulin resistance to be directly proportional to TSH and inversely proportional to adiponectin. Serum adiponectin, insulin sensitivity, and thyroid status might be linked to each other, which in turn may influence metabolic milieu and cardiovascular health through either disruption in lipemic equilibrium or other mechanisms. de Oliveira et al. (2019) found that T3 acts on the PI3K/Akt signaling pathway resulting in the differentiation of various cell lines, including adipocytes and thus resulting in increased adiponectin levels [27]. It also acts by enhancing the transcription of various adipokine genes. Thus, the administration of levothyroxine can compensate for the alteration and can maintain basal levels of adiponectin, which possess anti-inflammatory, anti-atherogenic, and insulin-sensitizing properties [17]. Though the effect of TSH on metabolic syndrome is interrelated to deficient adipokine formation, this association is not causal but additive and independent [28].

Serum hs-CRP has been considered a reliable biomarker for inflammation, which is recognized as a pivotal point in the pathophysiology of atherosclerosis, ischemic heart disease, and stroke and has a prognostic value on cardiovascular morbidity and mortality [29]. It showed a decreasing trend with therapy supporting evidence from other studies [30]. Serum adiponectin concentration at baseline is a good prognostic marker to differentiate between responders and non-responders with levothyroxine therapy and can help in classifying patients with good outcomes at initial assessments. The neural network algorithm can help to a great extent in predicting the risk of cardiovascular events with the help of the predictor covariates.

The elementary strength of the study lies in the fact that it has observed the holistic effect of levothyroxine therapy on anthropometric measurements, serum adiponectin, insulin resistance, and cardiovascular risk scores in an adequate number of patients. The relatively small sample size and observational study design were the potential limitations of our study. Randomized controlled trials are recommended for the results to be of a higher level of evidence and be generalizable to the population.

## Conclusions

In conclusion, thyroid status has an important effect on serum adiponectin levels, which may further be linked to insulin resistance and the cardiovascular health of the individual. Replacement therapy has a corrective effect toward the euthyroid, having corrective action on insulin sensitivity and risk indices predicting cardiovascular morbidity. This might be linked to the beneficial actions of adiponectin as an anti-inflammatory and anti-atherogenic agent. Moreover, a cardiovascular risk score can be predicted with the help of an artificial neural network with minimal errors using covariates such as age, gender, TSH, adiponectin, metabolic syndrome, and insulin resistance. These networks and further modifications can be applied to larger sample sizes to predict the scores more accurately.
